# Urotropin in Scarlatinal Nephritis

**Published:** 1909-03

**Authors:** 


### Urotropin in Scarlatinal Nephritis

A. Seibert, M.D., Professor of Pediatrics at the New York Polyclinic School and Hospital, reports (*New York Polyclinic Journal,* October, 1908), says he has used urotropin for the last two years with marked success, and relates his gravest case:

Female, age 11, became ill on April 2 with mild scarlet fever followed by multiple arthritis during second week of illness, and nephritis in third. May 12, two long attacks of severe uremic convulsions; during one of these, venesection. In preceding twenty-four hours four to five ounces of urine; albuminuria moderate. He took charge of case May 13, at the end of sixth week. universal edema, most marked in face; some ascites; marked pallor of mucous membranes; pulse, 48; threadlike. intermitting at every third beat; semiconscious; tongue covered with fungus; flat ulcerations in mouth with intense fetid oral odor; uremic amaurosis for five days; nausea and incessant vomiting even of water for ten days, necessitating rectal feeding.

In determining the cause of the extreme heart weakness, myocarditis was excluded by the fact that the child had outlived the two protracted convulsive attacks. The only alternative was that the uremic poison which had blinded the child was also depressing the nerve centers regulating heart action. Therefore all efforts were at once directed to the kidneys. Wet hot packs, colon flushing, diuretics and other measures had failed. To save the threatened life, the organisms which had invaded the kidneys had to be destroyed. Five gr. urotropin, dissolved in 1 ounce cold water, containing 15 drops dilute HCl, were given. Had this been vomited, a 2-gr. urotropin solution would have been given hypodermatically, though this has not been done hitherto: but the HCl stopped the vomiting. A small meal was retained.

The medication and a similar meal were repeated every three hours during daytime. Two enemata daily were given. Headache disappeared first, and marked diuresis set in, so that in the next twenty-four hours 110 ounces of urine passed. Edema began to subside after the second dose of urotropin, as did swelling in forty-eight hours, when the urine was free from albumin. Because of the HCl she got and retained appetite. Fever disappeared the second day. She began to distinguish objects after two days, and read without strain in a week: then the pulse also was normal. Four and one-half days after institution of urotropin she took a long journey for home. Within a week the 5-grain doses of urotropin were given three times instead of five times a day, and for another month twice a day. The formaldehyde in urotropin evidently produced a germicidal action in the infected kidneys.

